# Infantile hemangioma in a subadult Chinese pangolin: a case report

**DOI:** 10.1186/s12917-023-03832-9

**Published:** 2024-01-24

**Authors:** Xianghe Wang, Xuelin Xu, Fuyu An, Zhengyu Ren, Yongzheng Li, Kai Wang, Yan Hua

**Affiliations:** 1 College of Animal Science and Technology, Zhongkai University of Agriculture and Engineering, Guangzhou, 510550 China; 2 Guangdong Provincial Key Laboratory of Silviculture, Protection and Utilization, Guangdong Academy of Forestry, Guangzhou, 510520 China; 3 College of Life Sciences and Technology, Central South University of Forestry and Technology, Changsha, 410004 China

**Keywords:** Chinese pangolin, CT, Histopathology, Immunohistochemistry, Infantile hemangioma

## Abstract

**Background:**

Hemangiomas are a relatively common type of tumor in humans and animals. Various subtypes of hemangiomas have been described in the literature. The classification methods for hemangiomas differ between human and veterinary medicine, and the basis for tumor classification can be found in the literature.

**Case presentation:**

This study describes a tumor in the subcutaneous tissue of the right dorsum of an artificially rescued juvenile Chinese pangolin. Computed tomography (CT) examination yielded the preliminary diagnosis of a vascular malformation, and surgery was performed to resect the tumor. Histopathological examination showed that the tumor mainly was consisted of adipose tissue, capillaries, and spindle cells in the fibrous stroma. Immunohistochemistry showed the positive expression of CD31, CD34, α-SMA, GLUT1 and WT-1 in the tumor tissue, and the tumor was eventually diagnosed as an infantile haemangioma.

**Conclusion:**

The final diagnosis of infantile hemangioma was depended on the histopathological immunohistochemical and CT examination of the neoplastic tissue. This is the first report of infantile hemangioma in a critically endangered species Chinese pangolin.

## Background

The pangolin is the most trafficked mammal in the world. There are eight species of pangolin, and all listed as critically endangered by the International Union for Conservation of Nature (IUCN) [1]. Among these species, the Chinese pangolin (*Manis pentadactyla*) is mainly distributed in southern China and northern Southeast Asia [2]. The Chinese pangolin, belonging to the class Mammalia, order Pholidota, family Manidae, and genus *Manis*, is listed as a national first-level protected animal [1, 3, 4]. Due to their unique habits, habitat fragmentation, and illegal trade by humans, wild Chinese pangolin are on the brink of extinction [5]. Along with the decline of the wild pangolin population, artificial rescue and breeding is expected to be one of the effective means to protect this species [6, 7]. At present, research related to normal physiological indicators and diseases of pangolin is still lacking [6]. Some diseases such as dystocia, canine parvovirus and stomach ulcers, have been reported, but there are no published studies related to pangolin tumors [8–10].

This study describes a protruding tumor found in the subcutaneous tissue of the right dorsum of a rescued juvenile Chinese pangolin. The tumor was preliminarily diagnosed as a hemangioma by CT examination. Then we surgical excision of this tumor tissues using an ultrasound knife. Histopathology and immunohistochemistry were used to further analysis the excised tumor tissue, and the tumor was finally diagnosed as an infantile haemangioma.

## Case report

In this case, the Chinese pangolin (body weight: 1.65 kg) was rescued by the Guangdong Wildlife Rescue Monitoring Center. On September 26th, 2022, a raised lump was observed under the scales on the right dorsum; the lump felt hard upon palpation, and the skin showed no signs of redness, swelling, or ulceration.

**Fig. 1:**
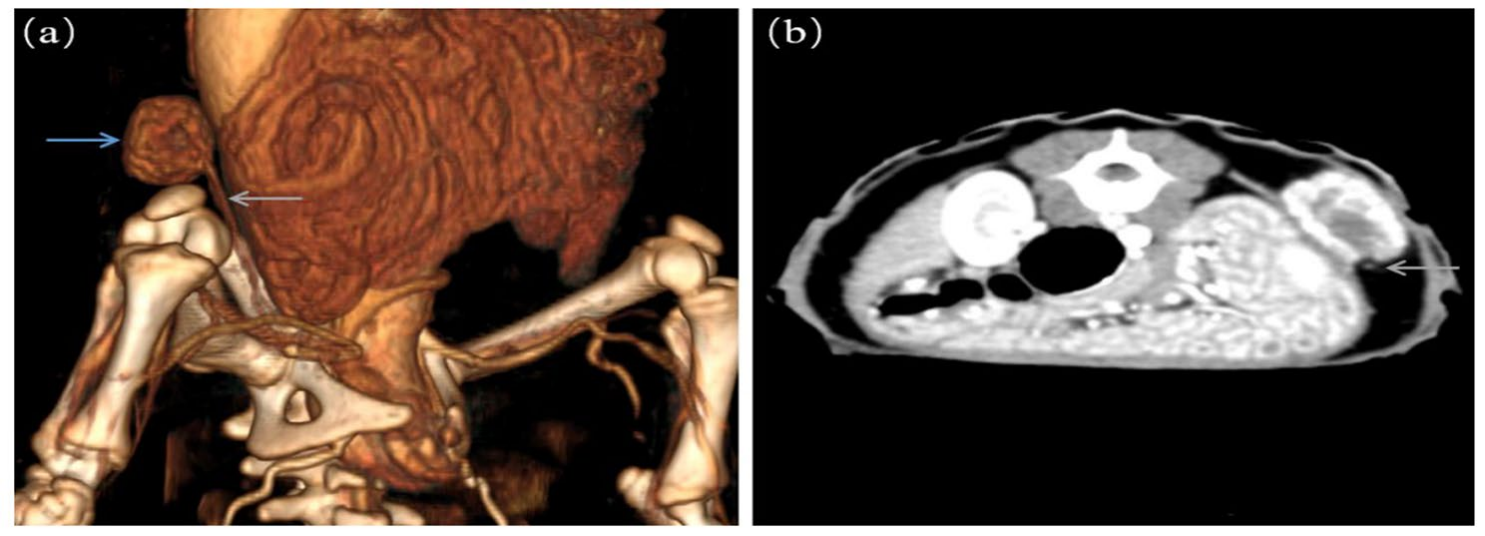
Imaging characteristics of the hemangioma in the Chinese pangolin. **a**. CT angiography 3D reconstruction; the blue arrow indicates the hemangioma, and the gray arrow indicates the blood supply vessel. **b**. 2D CT; the gray arrow indicates the blood supply vessel

The pangolin was placed in an inhalation chamber for anesthesia induction, and 5% isoflurane (100% isoflurane; Jiangsu HFQ Biotechnology Co., Ltd., Haimen, China) was circulated in the chamber with an oxygen flow rate of 2 L/min until the animal’s muscles relaxed. After anesthesia induction, anesthesia was maintained with 2% isoflurane in oxygen at 1.5 L/min through small mask and Mapleson type D nonrepetitive breathing circuit (Superstar Medical Equipment, Nan Jing, DM6A, China). A veterinary portable multiparameter monitor (Mindray, Guang Zhou, uMEC12Vet, China) was used to monitor the heart rate, body temperature, blood oxygen saturation, and ECG readings throughout the operation. After anesthetizing the animal, a CT (Min Found, ScintCare CT16, Hangzhou, China) scan was performed and a round heterogeneous enhancing soft tissue mass was observed under the skin of the right dorsum. The mass measured approximately 1.45 × 2.2 × 2.2 cm in size. Further intravenous injection of contrast agent (2 ml/kg iodohydrin, Fu’an Pharmaceutical Group Ningbo Tianheng Pharmaceutical, China) was performed, which revealed that the mass was supplied by a single blood vessel. The preliminary diagnosis was a vascular tumor (Fig. 1). On October 24th, another CT examination was performed and showed that the mass had grown to measure approximately 1.63 × 2.3 × 2.3 cm in size. The surgical procedures was conducted on November 12th, 2022 with an 8-hour fasting and 4-hour water restriction prior to the surgery.

Preoperative Routine blood biochemical testing (Mindray, BC-5000, automatic blood cell analyzer, Shen Zhen, China) showed that the pangolin had a higher white blood cell (WBC) count than those previously reported for Taiwanese pangolin (WBC count: 12.29 × 10^9^/L, reference interval [RI]: 3.50 ~ 11.2 × 10^9^/L), and amylase levels than those previously reported for Taiwanese pangolin (WMY count: 61 U/L, reference interval [RI]: 148 ~ 538 U/L) [11, 12]. These test results suggested that this pangolin had mild inflammation and mild dehydration. Dehydration may be related to fasting and water restriction prior to surgery. Other blood indexes showed no significant abnormalities.

**Fig. 2:**
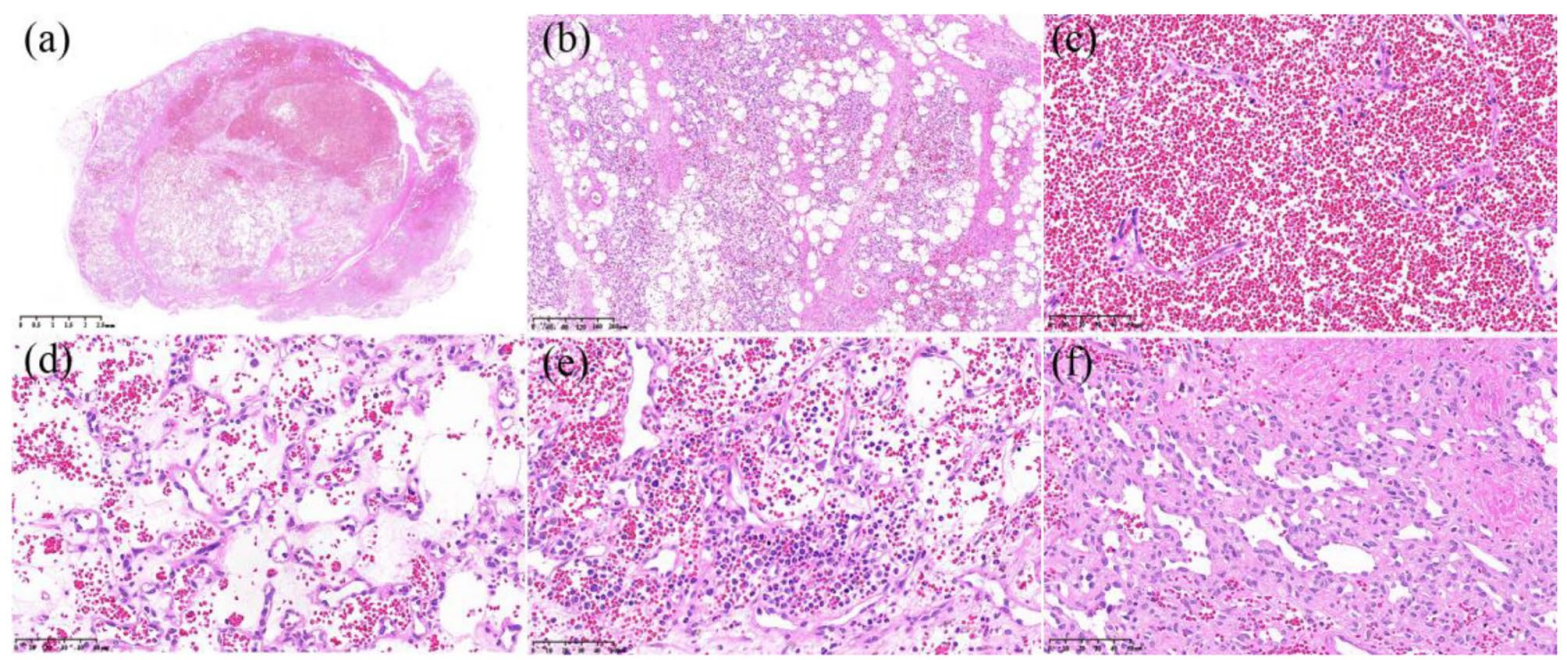
Histopathological staining results of the tumor. The mass was composed of multiple nodules with well-defined borders and no membranes (Fig. **a**, 1×), and the mass was nondestructively mixed with adipose tissue (Fig. **b**, 10×). Some regions of the mass were composed of numerous capillaries filled with many red blood cells (Fig. **c**, 40×). The capillaries were lined with endothelial cells with round to oval nuclei, and the interstitial components were not clear. Collagen fibers were dissolved, and there was interstitial edema (Fig. **d**, 40×). The interstitium was significantly widened, and many filamentous collagen fibers, as well as proliferated capillaries, were observed, along with many neutrophils in some local areas (Fig. **e**, 40×). The proliferative capillaries exhibited good differentiation, with little cellular atypia and few mitotic figures. In some areas, the tumor was composed solely of spindle-shaped cells in the fibrous stroma, and groups of these spindle-shaped cells were mixed with microtubular structures (Fig. **f**, 40×)

**Fig. 3:**
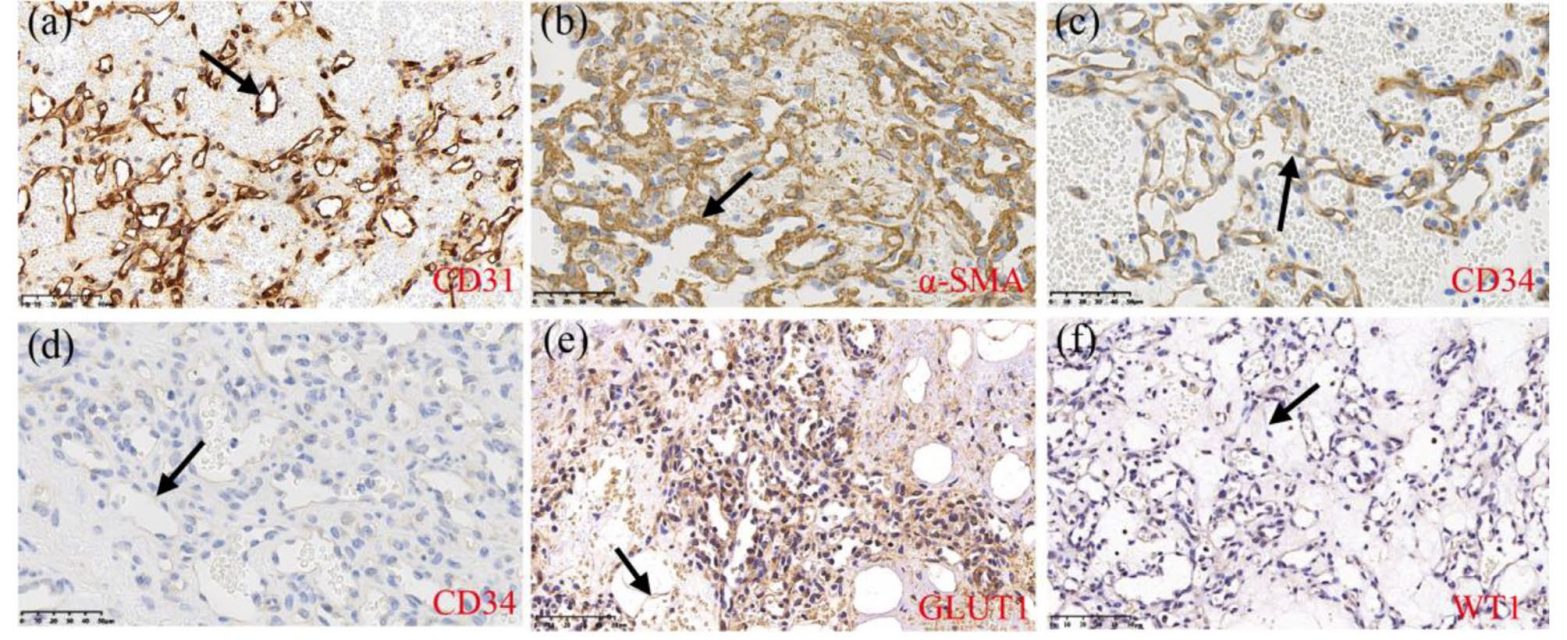
**ab**. Strong expression of CD31 and α-SMA in the cytoplasm of proliferating vascular endothelial cells (black arrows). **c**. CD34 expression only in capillary endothelial cells (black arrows). **d**. NO CD34 expression in the vascular lumen composed of spindle cells (black arrows). **e**. Weak GLUT-1 expression (black arrows). **f**. Weak WT-1 expression (black arrows)

A straight surgical incision approximately 2.5 cm long was made on the right side of the animal near the scales of the abdominal wall, away from the incision, tumor, which was accessed using blunt separation. The CT examination showed that the mass was fed by 3 vessels; thus, an ultrasonic knife (Super Veterinary Medical Technology Co.,Ltd., Shen Zhen, China) was used to excised the tumor. After the mass was excised, the subcutaneous muscular layer was closed with intermittent 4 − 0 monofilament nylon sutures, a drainage tube was placed, to facilitate postoperative wound flushing and prevent infection, and the skin incision was closed with intermittent 3 − 0 monofilament nylon sutures. The pangolin was injected subcutaneously with cefovecin sodium (8 mg/kg cefovecin sodium, Zoetis, Zoetis P & U, LLC, Kalamazoo, USA) once a day, and butorphanol (0.2 mg/kg Dolorex, MSD Animal Health Trading Co., Ltd. America) was administered once a day for postoperative analgesia. The injections were administered for 7 days. Additionally, the wound was washed with chlorhexidine solution daily. On the 31st day after treatment, the wound was healed, and there were no signs of recurrence at 5 months after surgery. Samples of the excised tissue was placed in nonbuffered formalin for fixation. After tissues were processed by dehydration. Tissue sections were stained with hematoxylin and eosin staining were performed and then examined with a light microscope (Leica DM1000, Leica Microsystems Trading (Shanghai) Co., Ltd.) observation. To further determine the tumor type, the prepared paraffin sections were dehydrated and antigenically repaired. This experiment immunohistochemical staining was conducted based on the EnVision System staining method. Endogenous peroxidase was then eliminated using 3% hydrogen peroxide (H_2_O_2_). Subsequent titration of goat serum (WuHan Boster Biological Technology Co., Ltd.) to block non-specific antigens. Then 100 µl of primary antibodies (α-SMA, dilution 1:500, Proteintech®), CD31 (dilution 1:1500, Abcam®), CD34 (dilution 1:1500, Abcam®), WT-1 (dilution 1:200, Servicebio®), GLUT-1 (Dilution 1:200, Servicebio®) were added to the tissue and incubated overnight at 4 ℃. To detect the expression of α-smooth muscle actin 100 µl of HCG-labeled secondary antibody (WuHan Boster Biological Technology Co., Ltd.) was added dropwise and incubated at room temperature for 2 h. Incubation at room temperature with drops of prepared diaminobenzidine (Genetech (Shanghai) Co., Ltd) colorant was followed by hematoxylin re-staining and observation after dehydration. Histopathological examination showed that the tumor nodes were composed of many vascular endothelial cells. There were a large number of red blood cells in the hyperplastic blood vessels, and the endothelial cells were flat and low in cell atypia, and their nuclear division was rare (Fig. 2). Immunohistochemistry showed strong positive expression of CD31 and α-SMA in the cytoplasm of proliferating vascular endothelial cells, CD34 expression only in capillary endothelial cells and not in the lumen of vessels composed of spindle cells, and weak expression of GLUT-1 and WT-1 (Fig. 3). Combined with the CT examination, the histopathologic and immunohistochemical results supported the diagnosis of infantile hemangioma.

## Discussion and conclusions

Currently, there are fewer studies related to tumors in Chinese pangolin. Stomach tumors of the pangolin were reported in 1984 and 1986 [13, 14], but the site of the tumour was later confirmed to be pyloric pillow of the stomach [15]. The National Forestry and Grassland Administration pangolin conservation research center is responsible for the artificial rescue of Chinese pangolin throughout the country. A pangolin with a hemangioma was found during our participation in the pangolin rescue, and the tumor was surgically excised. This is the first report about infantile hemangioma in sub-adult female Chinese pangolin. Surgical excision was performed, and no signs of recurrence were found on follow-up examination. This study provides reference materials for the diagnosis and treatment of diseases, particularly tumor diseases, in this endangered species.

At present, the classification of hemangiomas in animals is unclear, as well as the definition of each tumor subtype. The latest International Society for the Study of Vascular Anomalies (ISSVA) guideline classifies human benign hemangiomas as infantile hemangiomas, congenital hemangiomas, tufted hemangiomas, spindle cell hemangiomas, epithelioid hemangiomas, pyogenic hemangiomas, micro capillary hemangiomas and others [16]. In general, infant hemangiomas begin to grow at 2–3 weeks after birth, with 40 times more proliferating endothelial cells and mast cells in the tumor than in normal tissue. At the age of 5 years, 50% of infantile hemangiomas spontaneously reappear, the spontaneous regression rate significantly increased with age, and the spontaneous regression rate was 90% at 9.28 years old [17]. Spontaneous regression of hemangiomas has been reported in chickens, ducks and calves [18, 19]. Hemangiomas in animals are divided into capillary hemangiomas, cavernous hemangiomas and other mixed hemangiomas [20]. The tumor in this case was diagnosed as an infantile hemangioma based on CT, histopathological and immunohistochemical results.

CT was often used in the differential diagnosis of hemangioma and vascular malformation. The hemangioma in this case was supplied by a separate blood vessel and the findings on 3D reconstruction are consistent with the previous research [21]. The development of infantile hemangiomas can be divided into three phases: the proliferative phase, the stable phase and the receding phase. The histopathological examination of the specimen in this case revealed the dermal proliferation of lobularly arranged capillaries with flattened endothelial cells lining the capillaries, which is consistent with features reported in the literature [22–25]. The differential diagnosis of this tumor based on the histopathological and immunohistochemical results is demonstrated in Table 1. α-SMA was positively expressed in hemangioma tissue [26]. CD31 was positively expressed in vascular endothelial cells, and was negative expressed in cells of lymphatic [27]. In infantile hemangiomas, expression of the erythrocyte-type glucose transporter protein GLUT-1 is highly selective and diagnostic [28]. Additionally, the WT-1 gene, encoding GLUT-1, was expressed in the endothelium of hemangiomas but not in vascular malformations, which can be used to distinguish hemangiomas and vascular malformations [29]. The differential diagnosis of infantile hemangiomas and congenital hemangiomas can be made based on the fact that congenital hemangiomas are fully developed at birth. The histopathological results of this mass in this case were positive for CD31, CD34, α-SMA, GLUT-1 (glucose transporter protein-1) and WT-1 (nephroblastoma-1). Congenital hemangiomas and pyogenic granulomas are all negative for GLUT-1 [28–30]. In human medicine, infantile hemangiomas is are common benign tumors that can appear anywhere on the skin, but mainly develop on the head and neck [31]. Angiomas may be due to the mutation of gene related to angiogenesis in endothelial cells, resulting in an uncontrolled increase in angiogenesis [32]. In addition, increased estrogen can also induce the growth of hemangiomas [33]. The development of hemangiomas in dogs is thought to be associated with UV light exposure and sex [34, 35]. Further studies are needed on the pathogenesis of hemangiomas angiomas in Chinese pangolin.

The present study is the first published case of infantile hemangioma in a subadult female Chinese pangolin. We performed surgical excision of the tumor in this pangolin, and there was no sign of recurrence in the post operative review. This study fills a gap in the study of pangolin tumor and provides reference material for the diagnosis and treatment of tumor in endangered animals such as pangolin.

**Table 1:** Differential Diagnosis Table for Infantile Hemangiomas

Entity	Infantile hemangioma [28–30]	Congenital hemangioma [36]	Granulation tissue-type hemangioma [37]	Cavernous hemangioma [38]
Histopathology	-Proliferating capillaries consisting of flattened endothelial cells -Hyperplastic capillaries filled with red blood cells -Spindle cell populations mixed with microtubular structures	-Separated by fibrous tissue -Angiosarcoma consisting mainly of nested or whorled epithelial cells and bundles of spindle-shaped cells -Vascular lumen many not be clearly visible and consists of plump cells	-Small branching dilated capillary-type vessels -Massive vasodilation -Well-differentiated endothelial cells -Many red blood cells -Separated by fibrous tissue	-Hyperplastic capillaries filled with red blood cells -Proliferating capillaries consisting of a single layer of flattened spindle-shaped cells
Inflammatory cells	Neutrophilic granulocytes	Neutrophilic granulocytes, macrophages	Type and number of inflammatory cells depend on the development of the disease	Macrophages
Immunohistochemistry	Positive: GLUT-1, CD31, CD34, α-SMA, WT-1	Positive: CD31, Factor VIII Negative: α-SMA, cytokeratin	Positive: Factor VIII, α-SMA, vimentin Negative: Cytokeratin, desmin	Positive: CD31, cytokeratin AE1/AE3

## Abbreviations

kg: Kilogram
cm: Centimetre
CT: Computed tomography
SMA: alpha-smooth-muscle-actin
GLUT-1: Glucose transporter type 1
WT-1: Wilms tumor 1
